# Three-year mortality in previously hospitalized older patients from rural areas - the importance of co-morbidity and self-reported poor health

**DOI:** 10.1186/1471-2318-13-17

**Published:** 2013-02-19

**Authors:** Anne-Sofie Helvik, Knut Engedal, Geir Selbæk

**Affiliations:** 1 Department of Public Health and General Practice, Faculty of Medicine, Norwegian University of Science and Technology (NTNU), Postboks 8905, Trondheim, NO-7491, Norway; 2 Innlandet Hospital Trust, Division Tynset, Tynset, Norway; 3 St Olav’s University Hospital, Trondheim, Norway; 4 Centre for Old Age Psychiatric Research, Innlandet Hospital Trust, Ottestad, Norway; 5 The Norwegian Centre for Dementia Research, Oslo University Hospital, Ullevaal, Norway; 6 Faculty of Medicine, University of Oslo, Oslo, Norway; 7 Akershus University Hospital, Lørenskog, Norway

**Keywords:** Elderly, Gender, Hospitalization, Self-rated health, Survival analysis

## Abstract

**Background:**

The risk factors for mortality after hospitalization in older persons are not fully understood. The aim of the present study was to examine the three-year (1,096 days) mortality in previously hospitalized older patients from rural areas, and to explore how objectively and self-reported health indicators at baseline were associated with mortality.

**Methods:**

The study included 484 (241 men) medical inpatients with age range 65–101 (mean 80.7, SD 7.4) years. Baseline information included the following health measures: the Charlson Index, the Mini-Mental-State Examination, Lawton and Brody’s scales for physical self-maintenance and the instrumental activities of daily living, the Hospital Anxiety and Depression scale, self-reported health (one item), and perceived social functioning (one item) and assistance in living at discharge.

**Results:**

In all, 172 (35.5%) of those patients included had died within the three years of the follow-up period. Three-year mortality was associated with a high score at baseline on the Charlson Index (HR 1.73, 95%CI 1.09-2.74) and poor self-reported health (HR 1.52, 95%CI 1.03-2.25) in a Cox regression analysis adjusted for age, gender, other objectively measured health indicators, and perceived impaired social functioning.

**Conclusion:**

In a study of older adults admitted to a general hospital for a wide variety of disorders, we found co-morbidity (as measured with the Charlson Index) and poor self-reported health associated with three-year mortality in analysis adjusting for age, gender, and other health-related indicators. The results suggest that self-reported health is a measure that should be included in future studies.

## Background

Epidemiological studies with a follow-up time of between two years and twenty years have identified several health indicators as risk factors for mortality in general populations of older adults. These studies have included poor self-reported health [1-4], impaired cognitive function [5,6], impaired performance of the personal and instrumental activities of daily living [6-9], impaired vision and hearing [10], depression [8], and some chronic conditions [6,9,11,12]. Furthermore, it has been suggested that self-reported measures of health in general populations of older adults are equally appropriate for the prediction of mortality as more objective health measures [1-3,13], but not all the research agrees with this [14].

When it comes to studies exploring risk factors for mortality among hospitalized older patients with a wide variety of medical diagnoses, much the same objective health measures are reported as in studies of general populations of older adults, i.e. co-morbidity, reduced cognitive function, and impaired performance of the personal and instrumental activities of daily living, and depression [15-21]. The mortality is reported to be particularly high among hospitalized older adults with severe lung disorders [22], heart failure [23], stroke, and renal diseases [4]. In addition, the mortality risk after hospitalization among older patients has been found to be associated with having malnutrition at admission to hospital, the length of the hospital stay, and being discharged to assisted living after hospitalization [18-21,24]. Even though the follow-up time in these studies of mortality varied from six weeks [18] to 5 years [17], most of the studies had a one-year follow-up perspective [16,20,21,24-26]. Studies of the long- term mortality over three or more years among older adults from general medical hospitalized samples are rare [15,17]. Furthermore, to the best of our knowledge, studies of the risk factors for mortality after hospitalization in older patients have not included self-reported health measures.Thus, it is known that hospitalization of older persons is associated with long term increased risk of death, but the risk factors for mortality in older persons in need of hospitalization is not fully understood. The aim of this study was to explore if and how objectively measured and self-reported health indicators were associated with the three-year mortality in a sample of previously hospitalized older patients from rural areas with a wide variety of medical diagnoses.

## Methods

A prospective study with three-year follow-up was performed based on participants arriving at a general community hospital in Norway over a period of two years (1 September 2006 – 30 August 2008). The hospital included patients who came from nine rural municipalities.

### Participants

All the elderly patients 65 years and older admitted with an acute medical condition to the internal medical inpatients service, living in the region and hospitalized for at least 48 hours at the Tynset Division of the Innlandet Hospital Trust were considered for inclusion. Of the 802 possible participants in the study, 318 (40%) were excluded for the following reasons: severe cognitive impairment (116 patients), signified by a score of 3 on the Clinical Dementia Rating Scale [27,28]; severe communication difficulties (25 patients); being in a terminal state or having died before inclusion (47 patients); severely reduced general condition and physical functioning that made completion of the protocol impossible (mainly diagnosed as profound cardiovascular, pulmonary, or cancer conditions) (106 patients); or refusal to participate (24 patients) [29]. Thus, 484 patients were included and the follow-up time for each of them was 1,096 days (three years).

### Measures

Information about physical health (number of hospitalizations in the previous 5 years, length of stay, diagnoses, medications, and need for assisted living at hospital discharge) were obtained from medical records or hospital administrative systems. In Norway time of death is registered in Cause of Death Registry and transferred electronically to the hospital administrative system based on the unique national 11 digit identity number. Thus, as well information about time of death was collected from the hospital administrative system. Details of co-morbid diseases were collected using the Charlson Index [30,31] and employing Schneeweiss weighting according to the expected risk of mortality [32]. The Charlson Index is elevated when the score is higher than zero.

Cognitive function was assessed by means of the Mini Mental State Examination (MMSE), a 30-point interviewer-administered measure [33]. The MMSE has been translated, adapted and validated for Norwegian conditions [34]. The Clinical Dementia Rating Scale (CDR) assesses the severity of dementia, and a total score of 3 (range 0–3) indicates severe dementia [28].

The level of performance of the Activities of Daily Living (ADL) was measured by the Physical Self-Maintenance Scale (P-ADL, 6 items, score range 6–30) and the Instrumental Activities of Daily Living Scale (I-ADL, 8 items, score range 8–31) [35]. High scores indicate a lower level of performance for both scales. These questionnaires are used extensively in Norwegian studies of older persons [36,37]. The tendency to fall and the degree of vision/hearing impairment were self-reported employing single items from the population-based Health Study of Nord-Trøndelag [38] and Resident Assessment Instrument (RAI-AC) [39], respectively.

Anxiety and depression were assessed with the self-reported inventory Hospital Anxiety and Depression scale (HAD), which has seven items assessing depressive symptoms and seven items assessing anxiety symptoms (sum score 0–21 on each subscale). It has been developed to identify depression and anxiety in medically hospitalized patients [40]. High scores indicate more severe symptoms. The cut-off for depression (HAD-D) and anxiety (HAD-A) is set to ≥ 8 while the cut-off for having symptoms indicating at least a moderate disorder is set to 11 [41]. The HAD questionnaire has been validated in Norway and used in several studies including older adults [29,42,43].

Perceived health was measured by asking “How is your present state of health?” on a four-point response scale that included the following options “very good” (score 1), “good”, “fair” and “poor” (score 4) [44]. The scale was dichotomized as poor (score 4) versus not poor (score 1–3) in order to identify those with poor health.

Perceived social functioning was assessed by asking “To what extent have your physical health or emotional problems limited you in your social life during the last 4 weeks?” The five-point response scale ranging from “not limited at all” (score 1) to “not having any social life” (score 5) [38] was dichotomized in order to select those with impaired social functioning (score 2–5).

Socio-demographic information (living alone or not, smoking habits, and residence details) were self-reported employing the format of population-based health studies undertaken in Nord-Trøndelag [38,45].

### Procedure

All patients aged 65 years or older who were admitted to the department of internal medicine were assessed for inclusion in the study. Patients were invited to participate during their hospital stay as soon as they had been medically stabilized. The date and time of their inclusion were registered. The patients received written and verbal information about the study and, subsequently, gave their written consent. Initially, the research assistants administered the MMSE with all potential patients. If the MMSE score was 18 or lower, the CDR was performed. Those with severe dementia (CDR = 3) were excluded. Data were collected by two registered nurses (one specialized in geriatrics and one in health science) using a standardized procedure and interview. Prior to the start of the study, the nurses completed a two-day course on how to conduct the interview followed with training on a variety of healthy subjects. The inter-rater-reliability between the nurses was checked for the first 30 patients in the study and found acceptable (varying from 0.91 to 0.97).

The study was approved by the Regional Committee for Medical Research Ethics in south-eastern Norway and the Norwegian Social Science Data Service.

### Data analysis

Data were analyzed by means of the IBM SPSS, version 19.0. The sample characteristics were presented in two age strata because of the large age span covered in the sample. Descriptive analysis of independent samples was performed using the chi-square statistic or Fisher’s Exact Test for categorical variables (depending on the number of cases included). Independent sample *t*-tests or the nonparametric Mann–Whitney test were performed for continuous variables (depending on whether or not the distribution was normal).

Survival curves describing mortality during the 3 years after hospitalization were prepared by using Kaplan Meier plots for studying co-morbidity (yes/no) and poor self-reported health (yes/no) in analysis stratified by age and by gender and age. Log Rank (Mantel-Cox) was used to test differences in morality for co-morbidity and poor self-reported health by age and gender. A graphical inspection of the proportionality of the hazard assumption was carried out. Further, the Cox proportional hazard regression analyses were checked whether independent variables were time-dependent for the outcome. The proportional hazard was tenable (p-values > 0.3). No interactions between independent variables were found.

The main outcome, three-year mortality, was thus assessed by Cox proportional hazard regression analysis (unadjusted and adjusted) which takes into account both vital status and actual duration of survival. In the initial unadjusted analysis of three years mortality, the importance of gender, age, living status, smoking, the Charlson Index, the number of hospitalizations prior to inclusion, the duration of the hospital stay, the number of medications, MMSE, P-ADL and I-ADL, HAD-D, and HAD-A at T1, required assistance at discharge, as well as self-reported health, and social functioning were explored. The analysis of three-year mortality adjusted for age and gender with p-value ≤ 0.1 were subsequently included in two adjusted Cox proportional hazard regression models of three -year mortality (HR_A_, which included objectively measured health indicators, and HR_B_, which included health indicators both objectively measured and self-reported). P-values ≤ 0.05 were regarded as statistically significant.

## Results

### Sample characteristics

The study sample consisted of 484 patients, of whom 241 (49.8%) were men (Table 1). The mean age was 80.7 (SD = 7.4) years, with an age range of 65–101 years. The group of excluded patients had a similar gender distribution (55% women), but were older (mean age 82.8 years, SD 7.3, p < 0.001) and more of them died in the first six weeks after hospitalisation (12.9% vs. 5.6%, p < 0.001).

**Table 1:** Characteristics of study sample at T1by age (N = 484)

		**Total**	**Age**	
			**65-79 years**	**≥80 years**
	N (%)	484 *(100)*	207 *(42.76)*	277 *(57.23)*
**Socio-demographic**				
Age	Mean *(SD)*	80.66 *(7.43)*	73.64 *(4.28)*	85.91 *(4.29)*
Women	N (%)	243 *(50.21)*	94 *(45.41)*	149 *(53.79)*
Living alone	N (%)	248 *(51.23)*	80 *(38.64)*	168 (60.65)**
Smoking	N (%)	59 *(12.19)*	36 *(17.39)*	23 (8.30)**
**Medical information**				
Assisted living before hospitalization				
Nursing Home	N (%)	16 *(3.31)*	2 *(0.97)*	14 (5.05)**
Nursing care at home	N (%)	160 *(33.06)*	41 *(19.80)*	119 *(42.96)*
Domestic assistance at home ^a^	N (%)	58 *(11.98)*	13 *(6.28)*	45 *(16.25)*
No assistance or care	N (%)	250 *(51.65)*	151 *(72.95)*	99 *(35.74)*
Previous hospitalizations in last 5 years	Mean *(SD)*	2.16 *(2.61)*	2.33 *(2.92)*	2.03 *(2.35)*
Actual Hospitalization (days)				
Duration	Mean *(SD)*	6.42 *(5.21)*	5.94 *(5.10)*	6.74 (5.27)**
Duration before inclusion	Mean *(SD)*	4.33 *(3.55)*	3.82 *(2.92)*	4.72 *(3.92)*
Charlson Index	Mean *(SD)*	2.17 *(2.01)*	2.26 *(2.08)*	2.12 *(1.96)*
Main diagnosis on admittance				
Cardiovascular disease	N (%)	137 *(28.31)*	51 *(24.63)*	86 (31.05)*
Pulmonary disease	N (%)	101 *(20.86)*	55 *(26.57)*	46 *(52.71)*
**Impairment**				
MMSE	Mean *(SD)*	23.94 *(3.83)*	25.27 *(3.50)*	22.94 (3.77)**
P-ADL	Mean *(SD)*	9.01 *(3.46)*	8.13 *(3.41)*	9.66 (3.42)**
I-ADL	Mean *(SD)*	9.55 *(3.58)*	8.54 *(3.55)*	10.31 (3.42)**
Impaired hearing	N (%)	189 *(39.05)*	50 *(24.15)*	138 (49.82)**
Impaired reading vision	N (%)	109 *(22.52)*	35 *(16.91)*	74 (26.72)**
**Emotional situation**				
Prevalence of depression				
HAD-D ≥ 8	N (%)	50 *(10.33)*	20 *(9.66)*	30 *(10.83)*
HAD-D ≥ 11	N (%)	15 *(3.10)*	5 *(2.42)*	10 *(3.61)*
Prevalence of anxiety				
HAD-A ≥ 8	N (%)	45 *(9.29)*	23 *(11.11)*	22 *(7.94)*
HAD-A ≥ 11	N (%)	21 *(4.34)*	13 *(6.28)*	8 *(2.89)*
**Self-reported health**				
Perceived overall health as poor	N (%)	67 *(13.84)*	34 *(16.43)*	33 *(11.91)*
Perceived social function as impaired due to poor health	N (%)	336 *(69.42)*	137 *(66.18)*	199 *(71.84)*

*MMSE =* Mini Mental State Examination, *P-ADL =* performance of the personal activities of daily living, *IADL =* performance of the instrumental activities of daily living, *HAD-D =* The depression subscale of the Hospital Anxiety and Depression scale, *HAD-A =* The anxiety subscale of the Hospital Anxiety and Depression scale.

^a^ Assistance with cleaning, preparing food, shopping for groceries, etc.

^b^ Of those who scored *≤* 23 on the MMSE, there were 11 females and 13 males (total 24/484 = 5.0%) who scored <18 on the MMSE.

* p ≤ 0.05, ** p ≤ 0.01.

Table 1 shows the study characteristics of the sample divided into two age strata. Older adults, aged 80 and above, compared to those 65–79 years old, were more often living alone, smoked less often, had longer stays in hospital, lower cognitive functioning, lower P-ADL and I-ADL functioning, and more often suffered from impaired hearing and vision. In both age groups the two most frequent admitting diagnoses were cardiovascular disorders and pulmonary diseases, but a cardiovascular disease was more prevalent in the oldest age group.

### Mortality prevalence and health indicators associated with mortality

**Figure 1:**
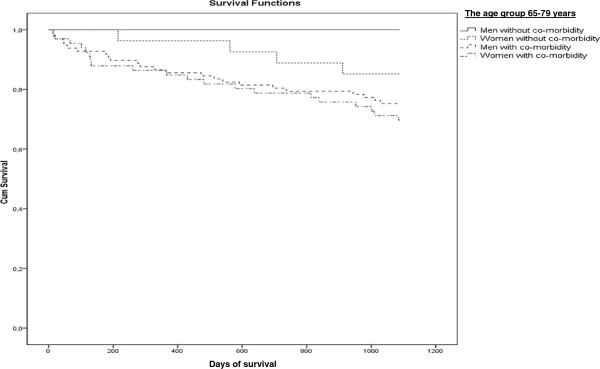
Kaplan-Meier plot: Days of Survival up to 3 Years (1096 days) in the Age group 65–79 years by Gender and with or without Co-morbidity (elevated Charlson Index or not).

**Figure 2:**
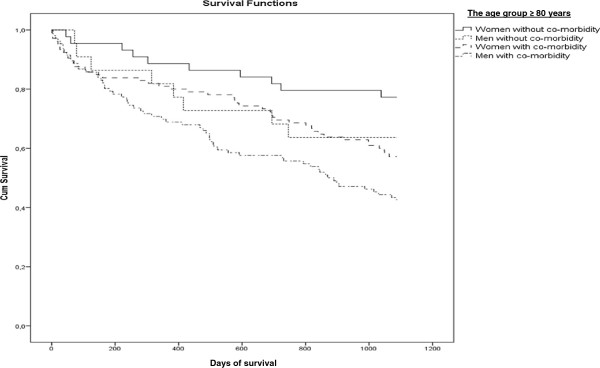
Kaplan-Meier plot: Days of Survival up to 3 Years (1096 days) in the Age group ≥ 80 years by Gender and with or without Co-morbidity (elevated Charlson Index or not).

**Figure 3:**
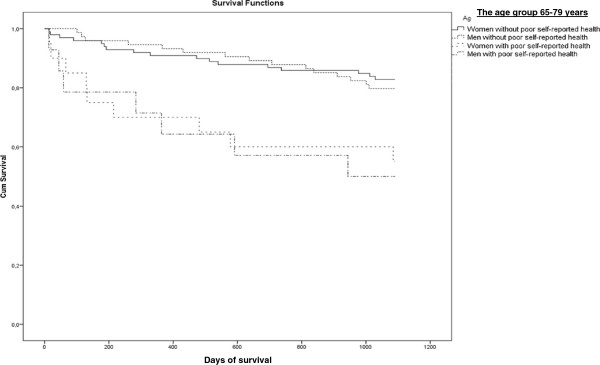
Kaplan-Meier plot: Days of Survival up to 3 Years (1096 days) in the Age group 65–79 years by Gender and with or without poor Self-reported Health.

**Figure 4:**
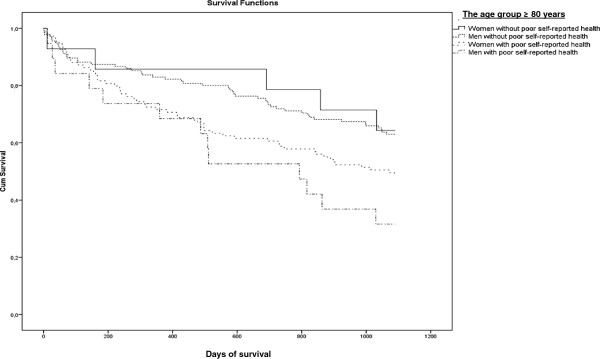
Kaplan-Meier plot: Days of Survival up to 3 Years (1096 days) in the Age group ≥ 80 years by Gender and with or without poor Self-reported Health.

In all, 172 (35.5%) of the included hospitalized patients had died within the three-year (1,096 days) follow-up period (Table 2). The mortality rate was described in two age groups by existence of co-morbidity and poor perceived health and gender in Figures 1, 2, 3 and 4. Significant mortality differences was found due to co-morbidity and poor self-reported health by gender and age by use of Log rank (Mantel-Cox) tests (Chi-Square 44.67 and 46.24, respectively; p < 0.01). The highest rate of death was seen among men in the oldest age group who had either an elevated score on the Charlson Index or poor perceived health.

**Table 2:** Mortality the first three years (1,096 days) after inclusion by age categories and gender (N = 484)

	**Age 65–79 years at T1**			**Age ≥ 80 years at T1**			**Total**
	**Women**	**Men**	**Total**	**Women**	**Men**	**Total**	
	**N (%)**	**N (%)**	**N (%)**	**N (%)**	**N (%)**	**N (%)**	**N (%)**
*Participants*	94 *(100)*	113 (100)	207* (100)*	*149 (100)*	128 (100)^a, b^	277* (100)*^*c*^	484* (100)*
*Mortality*							
Year 1	10 (10.64)	14 (12.39)	24* (11.59)*	*24 (16.11)*	37 (28.90)	62* (22.38)*	86* (18.18)*
Year 2	7 (7.45)	5 (4.42)	12* (5.80)*	*16 (10.74)*	16 (12.50)	32* (11.55)*	44* (9.09)*
Year 3	7 (7.45)	5 (4.42)	12* (5.80)*	*14 (9.40)*	16 (12.50)	30* (10.83)*	42* (8.68)*

T1 = baseline.

a = more men in the age group 80 years and over died during follow up than in the younger age group (Pearson Chi-Square =27.149, df3, p < 0.001).

b = more men than women in the age group 80 years and over died during follow-up (Pearson Chi-Square =9.784, df3, p < 0.05).

c = in all, more patients in the oldest age group died during follow-up than in the youngest age group (Pearson Chi-Square = 23.356, df3; p < 0.01).

In separate Hazard ratio analysis adjusted for age and gender both reduced health from objective health measures and self-reported measures were associated with increased of mortality, i.e. elevated Charlson Index (HR 2.15, 95%CI 1.37-3.37), number of hospitalizations last five years (HR 1.09, 95%CI 1.03-1.14), duration of hospitalization (HR 1.04, 95%CI 1.01-1.07), MMSE ≤ 24 (HR 1.64, 95%CI 1.17-2.30), P-ADL > 6 (HR 2.66, 95%CI 1.70-4.17), nursing care at home or in institution compared to no assisted living after hospitalization (HR 2.19, 95%CI 1.17-2.43), poor self-reported health (HR 1.94, 95%CI 1.33-2.82) and restricted social functioning due to poor health (HR 1.68, 95%CI 1.17-2.43), respectively. In hazard ratio models of mortality, the objectively measured health indicators (the Charlson Index, the number of hospitalizations before inclusion, and impaired personal functioning at inclusion) were independently associated with increased three-year mortality in analyses adjusted for age, gender, cognitive and instrumental functioning, duration of hospital stay, and need for assisted living at discharge (Table 3). In the fully adjusted hazard ratio model including both objectively measured and self-reported health indicators, co-morbidity measured by an elevated score on the Charlson Index and poor self-reported health were associated with an increased three-year mortality risk.

**Table 3:** Hazard ratio for three-year mortality in adjusted analysis (N = 484)

	**HR**_**A**_**(95% CI)**	**HR**_**B**_**(95% CI)**
*Objectively measured health indicators at T1*		
Elevated Charlson Index	**1.79 (1.13-2.83)**	**1.73 (1.09-2.74)**
Number of hospitalizations last 5 years	**1.06 (1.01-1.14)**	1.05 (0.99-1.68)
Duration of hospitalization	1.02 (0.99-1.05)	1.02 (0.99-1.05)
MMSE ≤ 24	1.29 (0.90-1-84)	0.76 (0.53-1.09)
P-ADL > 6	**1.72 (1.04-2.84)**	1.59 (0.95-1.07)
I-ADL >8	1.13 (0.78-1.64)	1.16 (0.80-1.68)
No assisted living after hospital stay	1.00 Reference	1.00 Reference
Domestic assistance at home ^a^	1.24 (0.69-2.24)	1.26 (0.70-2.27)
Nursing care at home or in institution	1.45 (0.94-2.23)	1.39 (0.90-2.15)
*Self-reported health indicators at T1*		
Self-reported health seen as poor		**1.52 (1.03-2.25)**
Self-reported impaired social function due to poor health		1.17 (0.79-1.72)
−2 Log Likelihood	1941.87	1934.18

*T1*, Baseline.

*HR*, Hazard ratio.

*CI*, The confidence interval.

_A_ The Cox proportional hazard regression analysis where each regressor was adjusted for increasing age, gender and the other variables in the model.

_B_ The Cox proportional hazard regression analysis where each regressor was adjusted for age, gender and the other variables in the model.

^a^ Assistance with cleaning, preparing food, shopping for groceries, etc.

*MMSE*, Mini Mental State Examination, *P-ADL*, performance of the personal activities of daily living, *IADL*, performance of the instrumental activities of daily living.

## Discussion

To the best of our knowledge, this is the first three-year mortality study in the Nordic countries among older medically hospitalized adults with a wide variety of medical diagnoses assessing the importance of both objectively measured and self-reported health indicators for the risk of death. In an adjusted Cox regression analysis, three-year mortality was associated with co-morbidity (an elevated score on the Charlson Index) and poor self-reported health.

Among the variables included in this study, co-morbidity was found to be the most evident risk factor for three-year mortality. The mortality risk was almost doubled when the Charlson Index was elevated. The result of the study was not unexpected and in line with the results of other studies. Co-morbidity has been found to be important for mortality in a one, two, three or five year perspective among previously hospitalized older patients [16,17,19,25,26]. In the adjusted Cox regression model of objectively measured health indicators, the number of hospitalizations in the five years before inclusion and impaired performance of the personal activities of daily living at inclusion were independent risk factors for death. Impaired performance of the personal activities of daily living has commonly been found to be associated with increased risk of death among previously hospitalized older patients, and thus our results were not surprising [16,17,19,21,25,26]. In the same model, we did not find impaired cognition (MMSE ≤ 24) to be associated with an increased three-year mortality risk. Some hospital studies have found that reduced cognition is associated with one-year [21] and long-term mortality [15,17], but not all have reported such an association [16,26]. Furthermore, some studies of one-year mortality have found increased mortality in those with assisted living after discharge [21,24], and a longer hospital stay [21], but not in all [16]. Internationally, the length of hospital stay and admission to assisted living will vary not only due to health indicators such as co-morbidity and impaired personal ADL function in the patients, but also due to different cultures of care and the financial circumstances of the countries concerned.

In contrast to other studies of mortality in older previously hospitalized elderly patients, we explored whether self-reported health indicators were associated with three-year mortality when adjusting for objectively measured health indicators, age, and gender. Our results showed that self-reported poor health was related to an increased risk of long-term mortality, which is quite consistent with results from epidemiological studies of older adults living in the community, even when the analyses had been adjusted for objective health indicators [1]. In accordance with what has been suggested in the epidemiological studies, the reason for our finding may be that perceived health gives more or additional (in the present study) information than the objectively measured health indicators alone [1]. Self-reported health incorporates both peoples’ subjective assumptions and their actual knowledge of their own health status [1,46,47]. Furthermore, self-reported health may be an expression of a more complex judgment about health status than the objectively measured indicators, as this concept may include to how adequately a person can cope with their own health situation [1,48].

This study has some limitations. Firstly, the most severely ill patients, both in terms of cognitive function (severe dementia or delirium) and physical health, were not included. Therefore, the study is not representative of all older adults admitted to a hospital stay. An inclusion of the most fragile patients would certainly have increased the mortality rate and could have led to other results, but to include the most frail group with impaired competence to consent by using information from proxy persons was difficult in the hospital setting and might have led to new methodological considerations and problems in evaluating the results [29].

Secondly, the hospitalized patients in our study came from rural areas of Norway. Even so, there is no reason to believe that the risk factors for mortality in previously hospitalized older persons due to somatic health problems are different in rural than they would be in urban areas in the Nordic countries [26]. In general populations of older people in rural areas, risk factors for mortality do not seem to be different from other general populations of older persons [8].

Thirdly, hospitalized patients often have reduced capacity to fill in long questionnaires. Therefore, we assessed perceived health by a single item, as many other studies have done. Idler and Benyamini [1] reviewed almost thirty studies that used a single question to rate self-reported health. The wording of the single item varied between the studies and it is debatable how well each of them tapped such a complex and multidimensional phenomenon as health. The question we used has been used frequently in other studies and found to be sensitive in epidemiological studies by other Norwegian research groups both in community and hospital settings [44,45,48,49]. In addition, the question we used has been found preferable in clinical studies of mortality compared to other measures of self-reported health [2].

Fourthly, the Charlson Index was used to score co-morbidity. However, one could argue that, in addition, we should have added the main reason for hospital admission (diagnosis) in our analyses, which has been done in some other studies [21]. Unfortunately, we did not have the statistical power to carry out such analyses.

Fifthly, we have not included information of other known possible risk factors of mortality such as the patient’s nutritional state [16,18-20,24], living accommodation, and social support prior to hospitalization [20]. We did not have access to such data and, therefore, we cannot know whether such information would have influenced our results, and the subject remains open for discussion. Lastly, even though we used a longitudinal design, direct causality cannot be inferred from the associations that we have demonstrated.

## Conclusion

In a study of older adults admitted to a general hospital for a wide variety of disorders we found that co-morbidity as measured with the Charlson Index and poor self-reported health increased the risk for three-year mortality in analysis adjusting for age, gender, and other health indicators. The results suggest that self-reported health is a measure that should be included in future studies. The deeper meaning of this concept merits closer investigation.

## Competing interest

There are none competing interests.

## Authors’ contribution

ASH has participated in design of the study, analyzed the data, participated in interpretation of results and drafted the manuscript. KE and GS has participated in the design of the study, the interpretation of study results and editing the manuscript. All authors read and approved the final manuscript.

## Pre-publication history

The pre-publication history for this paper can be accessed here:

http://www.biomedcentral.com/1471-2318/13/17/prepub
